# Boston Letter

**Published:** 1880-05

**Authors:** 


### Domestic Correspondence.

### ARTICLE X.

### BOSTON LETTER.

MESSRS EDITORS: For a third time I touch upon the admission of women to the Massachusetts Medical Society. The opinion already expressed, that the matter is not by any means settled, received full indorsement at the February meeting of the councilors of the society. The protest of the censors against the October action of the councilors, and to which I referred in my last letter, was presented at the late meeting and convinced the councilors that they had indeed overlooked a vital point, the gist of which I have already explained. It was found that the present society must be consulted, perhaps the legislature as well. A motion to refer the matter to the general society was overborne by the wiser suggestion that the subject should receive more careful consideration in the council. The October vote, which was in favor of the admission of women to the society, was reconsidered, the opposition to this course being notably weak. The whole matter, therefore, now stands where it did before it was introduced to the council. It probably will give rise to a warm discussion at the June meeting of the councilors. In the course of time the subject will be brought before the main society, which, unless I am much in error, will finally shelve it.

The chief bone of contention at the last meeting of the council, was the code of ethics. Two codes were presented. One was the majority code, signed by four of the committee of five. It had once before been submitted to this body, and at that time was roughly handled, and then re-committed to the committee which was now increased by two members, one of whom was Dr. H. J. Bigelow. During the interval between the meetings of the councilors, Dr. Bigelow took the majority code, pruned and condensed it into the form finally accepted by the council. This was the minority code. Its text has been published in the Boston *Medical and Surgical Journal*, and at the present time is being discussed by the medical journals throughout the country. The contention to which the two codes gave rise in the council brought forth a curious diversity of sentiment. The majority of members from the country favored the acceptance of the long code with its unnecessary detail, and its suggestions of unethical sloughs, the existence of which was so far removed even from the imaginations of many of the councilors, that they indignantly repudiated the necessity of mentioning them. "If," said one gentleman, "members of this society have not the instincts of courtesy and honor, this code will never influence them." "I have been a physician for thirty years," said another, "and until I read this code, supposed I was one of a body of gentlemen. I cannot believe that the practices suggested by the majority code ever existed," etc. The friends of this code felt that elementary instruction in ethics was a paramount necessity to young as well as to many old physicians. Their tone of thought reminded one of the woman who on leaving her children alone, told them not to put beans in their noses, which all of them straightway did so soon as her back was turned, though they never had thought of the trick before. Perhaps the equally unnecessary suggestion in the majority code would have created a like result.

The friends of the minority code justly felt that the mere idea that such puerile instruction and such hints of evil practices as were contained in the majority code were necessary to a liberal profession, was almost an insult. Others there were who felt that since the society had existed in peace for nearly one hundred years without a code, there was no need of one at the present day. But they were willing to fall in with the majority in the acceptance of the concise, sensible minority code, their feeling being very neatly expressed in a recent editorial written by Dr. Geo. B Shattuck: "To the members of the profession in Massachusetts, we wish a pleasant journey under the auspices of this compact little code, and hope it may prove a pillar of fire to the ignorant Israelite and a Red Sea to the false Egyptian."

In behalf of Dr. Cotting who was the active author of the majority code, it should be said that but for the latter the minority code, in all probability, would not have made its appearance. One is the basis of the other.

For several weeks the medical and lay public have been agitated over the proposed act to regulate the practice of medicine. The bill was the outcome of the discussions in the Social Science Association. After being submitted to the legislature, a committee was appointed whose duty it was to listen to evidence in favor of and against the bill. Very curious, very amusing and very grave developments were the result. The opponents of the bill were charlatans of every ilk. Long-haired spiritualists and clairvoyants of the male sex and unwholesome females of doubtful occupation; fanatics and selfish individuals of every stage of ignorance, appeared as remonstrants. So forbidding were they in mien—so ignorant and unblushing in their arguments, that it was felt that no other testimony in favor of a medical code was necessary. The bill in question united regular, homœopath and eclectic physicians. A board of examining censors, which should represent each of these bodies, was to be formed. Applicants for licenses to practice were to be required to give evidence of a fair medical education, and to present a diploma of graduation from a legalized school. Therapeutics were to be tabooed, so that no point of difference could arise between the censors. In short, the object of the bill was to rid the State of its horde of untaught, dishonest quacks. We are overridden by them. They are a sore upon the body politic—a festering excrescence—a band of thieves. The petition which accompanied the bill, upon its presentation, was signed by very many of our most highly educated and wisest regular physicians. At the outset, the feeling that the bill should pass the legislature—that it was a dire necessity—was very general. Even those who did not agree with all the clauses of the medical act, felt that it would be better to have this bill made a law, immature as it was in some respects, than to permit quacks any longer to abuse the confidence of the credulous. It would be a simple matter to modify the act at a later day. On the other hand, many members of our regular State society opposed the bill because it recognized homœopaths and eclectics, and opened the way to an undesirable affiliation between them and the regular school, giving the three bodies a common position before the public, as well as equal standing and equal rights. Another objection was that the bill gave the governor and council the power of appointing the censors, instead of leaving it where it should be—in the hands of the proper and only competent party, the medical profession itself. Another objection was that such a bill could no more be enforced than a liquor law. Opposition also arose on the ground that no matter how carefully a bill may be drafted, it will be unsuccessful, because the people will employ quacks, even while knowing that they are so. And so on. You can see how objections might be multiplied. The bill, however, was defeated. A minority medical act was then presented to our general court, and that likewise suffered defeat. So that we not only have no law, but the quacks are more than ever firmly entrenched. But one endeavor to regulate the practice of medicine having been fairly made, and public thought in regard to this matter having been awakened, further, and let us hope more successful, efforts probably will be made to protect the State against the knavery of the swarm of charlatans now at work among us.

The homœopaths are making strenuous attempts to gain admittance, in a professional capacity, to our city hospital, which thus far has been conducted by the best representatives of the regular school. Since the hospital is a city institution, supported by funds drawn from the city treasury, and therefore dependent upon taxpayers who do, as well as upon those who do not, employ regular physicians, it can hardly be said that homœopaths have no right to demand representation within its walls. Their *pro rata* rights, however, are very diminutive. This matter is not yet settled. Indeed, it is merely in embryo. The homœopaths would like to develop a strong opposition to their desire to secure wards in this hospital. In fact, they court this opposition. An editorial in a recent number of the *New England Homœopathic Gazette*, after kindly speaking of members of our school as "self-conceited bigots," makes the claim that opposition has always aided the homœopathic fraternity, and adds that still more resistance to them on the part of the regular medical faculty would be a good thing. Judging from the spirit of this editorial, a layman would actually think we persecuted homœopaths, when the fact is that one rarely finds a homœopathic journal without its bitter attack on the regular profession. On the other hand, the journals of the regular school seem to have made a tacit agreement to avoid all but strictly necessary mention of the so-called homœopaths, who, with some very rare exceptions, are so untrue to the Hahnemann faith that they do not deserve the name they wear. * *

BOSTON, April 14, 1880.

---

CONGENITAL NUCLEAR CATARACT.—Alfred Græfe called attention, at the Ophthalmological Congress at Heidelberg, to a form of nuclear cataract of very firm consistence, which is found in infants, but which, although probably not unfrequently met with, has, in his opinion, not received sufficient notice in the literature of cataract. It is, however, of importance with reference to the proper means to be adopted for its removal. On attempting to needle this form of cataract its density becomes evident from the movement imparted to the lens. The result of the decision is also unsatisfactory, imbibition and absorption taking place only imperfectly. The general opinion of those members who took part in the discussion following the paper was that extraction is the proper method of treating such cases, and that this operation can safely be performed on very young children. In the January number of the *Centralblatt f. Augenheilkunde*, Dr. Just publishes seven successful cases of extraction in infants, in which cases the cataract was of hard consistence. No bandage was used after the operation. He believes that this form of cataract is not congenital, but begins very shortly after birth. This opinion seems, however, to be based entirely on the statements of the mothers.

